# Methodological approach to spatial analysis of agricultural pest dispersal in olive landscapes

**DOI:** 10.1007/s10661-022-10068-x

**Published:** 2022-05-09

**Authors:** A. Moreno, A. J. Rescia, S. Pascual, M. Ortega

**Affiliations:** 1 Department of Biodiversity, Ecology and Evolution, Faculty of Biological Sciences, Complutense University of Madrid, C./ J.A. Novais, 12, 28040 Madrid, Spain; 2 Department of Plant Protection, National Institute for Agricultural Research and Experimentation, Carretera de La Coruña (A6), km. 7,5, 28040 Madrid, Spain

**Keywords:** *Bactrocera oleae*, Cost distance tool, Landscape permeability, *Prays oleae*, Pest movement, Smart farming

## Abstract

The effectiveness of a Geographical Information Systems cost-distance tool for detecting landscape permeability in relation to the movement of pests in olive landscapes was established. The simplification of agricultural systems is linked to an increased incidence of pests on crops. Therefore, it is important to understand the impact of different land uses surrounding olive groves on pests. In this work, we analysed the effect of the structure of the olive landscape on the movement of two main olive pests—the olive fruit fly, *Bactrocera oleae* (Rossi) (Diptera: Tephritidae) and the olive moth, *Prays oleae* (Bernard) (Lepidopetera: Praydidae). We applied linear mixed effects models to analyse the relationship between pest abundance and cost-distance, using different hypotheses to evaluate those land uses that are favourable or unfavourable for the movement of these pests. The results show that this methodology is effective in detecting possible unfavourable land uses with a barrier effect, such as woodland and artificial land uses, and favourable land uses with a corridor effect such as olive groves. Whether other land uses, such as scrubland or riverbanks, act as a barrier or corridor depends on the pest and its life cycle stage. The effect that different land uses have in maintaining low levels of pest populations and ensuring the long-term sustainability of these agricultural systems are discussed. The implications of landscape permeability for the physical structure of the landscape and the dispersal of organisms, and the potential of that landscape to impact the continuous flow of natural processes are also addressed.

## Introduction

One of the major concerns of the agricultural sector is the increase in productivity beyond the increase in production (yield) through more intensive use of inputs. Productivity is related to changes in technology, efficiency, management capacity and organisation of production (EU, 2018). In recent decades, attempts to achieve higher productivity of agricultural systems, through the expansion of monocultures and intensification of management, have led to a spatial simplification in agricultural landscapes where crop yields and productivity have increased (Bianchi et al., 2006; Foley et al., 2005; Tscharntke et al., 2005). This has led to an increase in provisioning ecosystem services to the detriment of other ecosystem services, as well as a significant loss of biodiversity (Cardinale et al., 2012; Zhang et al., 2007). One of the direct consequences of these spatial and ecological changes has been the increased ease of arrival and spread of pests, which has led to significant economic losses in the agricultural sector (Rusch et al., 2016). To mitigate this damage, producers often resort to the use of phytosanitary products, which is associated with soil and aquifer contamination or pollinator decline, among other problems (Meehan et al., 2011; Potts et al., 2010; Vanbergen & Initiative, 2013). Therefore, management practices that are considered more environmentally friendly, such as conservation biological control, have been gaining acceptance as more sustainable and efficient tools in the long term (Bianchi et al., 2006; Jonsson et al., 2015; Paredes et al., 2013).

As already mentioned, the increase in agricultural productivity is linked to technological changes that improve farm management. Undoubtedly, the digitisation of agriculture (i.e. smart farming transition) that has occurred in recent years is aimed at increasing yields, and therefore profitability, by reducing or controlling the cost of inputs (Bacco et al., 2019). Specifically, the aim is to improve the energy efficiency of crops through the optimisation of two essential aspects for production—irrigation and pest control methods, the deficiency of which causes the greatest economic losses for farmers. In this sense, the use of computer tools can greatly support efficient agricultural management. Thus far, most studies related to conservation biological control in agricultural systems at the landscape scale have focused on the potential presence of natural pest predators in the surrounding areas. However, studies focusing on pests, both their biology and their relationship to the landscape, are much less common (Mazzi & Dorn, 2012; Sivakoff et al., 2012). One approach to this problem has been the exploratory studies of landscape composition, in which relationships have been established between the areas of each type of land use and the population density of taxa (Prasifka et al., 2004; Rusch et al., 2016; Schmidt et al., 2008). However, there is a lack of tools that indicate the physical infection capacity of pests, i.e. their spatial range. This parameter depends on their abilities to disperse through the different spatial patterns surrounding olive groves.

In the last few years, monitoring and predictive models in the field of geographic information and remote sensing have been improved using Geographical Information Systems (GIS) (Conolly & Lake, 2006; Van Leusen, 2002). This is the case for the study of agricultural landscapes, where these systems are acquiring greater relevance for understanding complex metrics such as interaction networks, spatial connectivity and pest migration routes. An example of this is the tool developed by the geospatial geoprocessing software ArcMap called “Cost-Distance”. This tool employs an algorithm based on the node/link cell representation, used in graph theory, to assign different cost values to the cumulative displacement of an organism from a given origin to any destination point (ArcGis Desktop, 2020). In agricultural landscapes, the Cost-Distance tool has been used in numerous research related to connectivity and landscape architecture (Adriaensen et al., 2003; Richard & Armstrong, 2010). Initially, one of the most common uses was as an index to establish and delimit links between patches of natural vegetation useful for wildlife (wildlife linkages), as well as to study gene flow between spatially separated populations of the same species (Beier et al., 2008, 2009; Coulon et al., 2004). A more recent application is exemplified in a study carried out by Perović et al (2010). These authors studied how populations of different natural enemies of cotton pests fluctuated in Australia based on the influence exerted by the landscape. Specifically, they identified the land uses that were more or less favourable to natural enemies, as well as the scale at which they were most effective for biological control of cotton pests. Other studies have applied this same tool to determine the effect that the environment, understood as the different land uses, can have on the dispersal capacity, distribution and population density of a given taxon. In this case, technically, the cost map generated represents the effort/difficulty involved for that taxon to reach each cell of the grid, or raster, in which the territory has been defined (Driezen et al., 2007). This new holistic view, where the landscape is considered as a whole, allows us to analyse in an objective and functional way the influence exerted by each of the elements that compose it (land uses), in combination with each other, on the behaviour and phenology of the taxon under study.

In the present study, the landscape considered is very typical of Mediterranean environments and comprises large areas of olive monoculture. In recent decades, these agricultural landscapes have seen an increase in damage caused by its two most important pests, *Bactrocera oleae* and *Prays oleae* (MAPAMA, 2014). Numerous studies have been carried out to better understand their effects on olive cultivation, and their phenology and interaction with the environment (Boccaccio & Petacchi, 2009; Ortega & Pascual, 2014; Ortega et al., 2016; Paredes et al., 2019; Plata et al., 2019; Villa et al., 2020). However, the role the different land uses in the area play in the spread of these pests and their degree of influence expressed as risk of spread are unknown. The objective of this work is to analyse the influence of different types of land use on the movement of the pests *Bactroecera oleae* and *Prays oleae.* This analysis will be based on hypotheses of different costs in each land use that can be contrasted with real data on the abundance of these pests in olive-growing areas in southern Spain. In this way, it would be possible to identify those land uses that are more or less favourable to the spread of pests to curb their expansion in these agrosystems. In our study, the aim is to quantify the permeability of the landscape, understood as a measure of landscape structure with some land uses as barriers and other as corridors. This serves as an indicator of the quality of a landscape to provide passage for different types of organisms (Anderson & Clark, 2012; Singleton et al., 2002). A total of 18 hypotheses were developed, each with a different resistance value assigned to the identified land uses. In addition, an analysis was carried out at different spatial scales to determine the risk of species spread throughout the territory. Specifically, the aim was to: (i) identify the barrier or corridor effect of each of the different land uses in the dispersion of pests in the olive landscape, using the different hypotheses where land uses change from favourable to unfavourable values; (ii) establish the spatial scale at which barrier or corridor effects of land uses on pests can best be detected, therefore indicating the appropriate scale for landscape composition planning.

## Materials and methods

### Study area

**Fig. 1:**
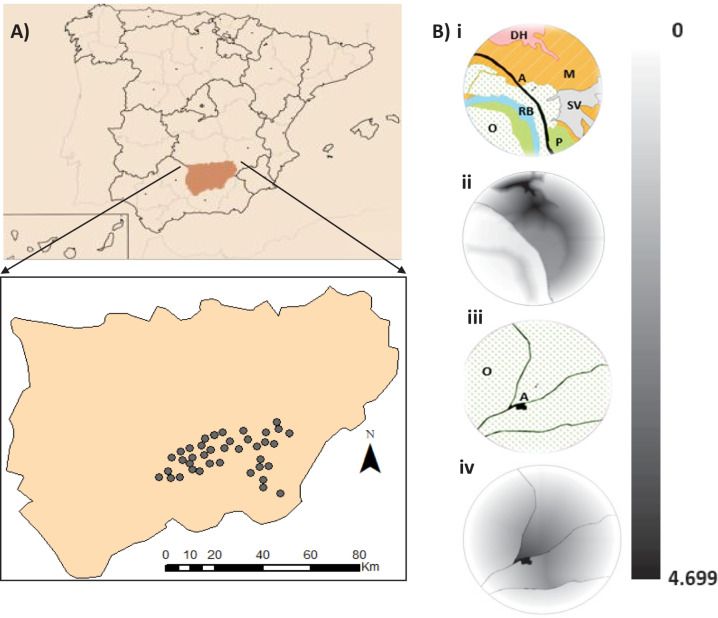
**A** Geographical location of the study area and detailed view of the spatial distribution of the 39 sampling points located in the province of Jaén, Andalusia, Spain. **B** Example of circular areas with their lands uses and the results of the application of Cost-Distance tool for hypothesis 16 at sampling points representing a complex landscape surrounding the sampled point (i) and representing a simple landscape with only olive groves and artificial infrastructures (iii), obtaining cost layers (ii) and (iv) respectively. Darker shades (4.699, maximum) denote higher resistance. Acronyms indicated by artificial (A), *dehesas* (DH), olive grove (O), grassland (P), riverbank (RB), sparse vegetation (SV) and scrublands (M)

**Fig. 2:**
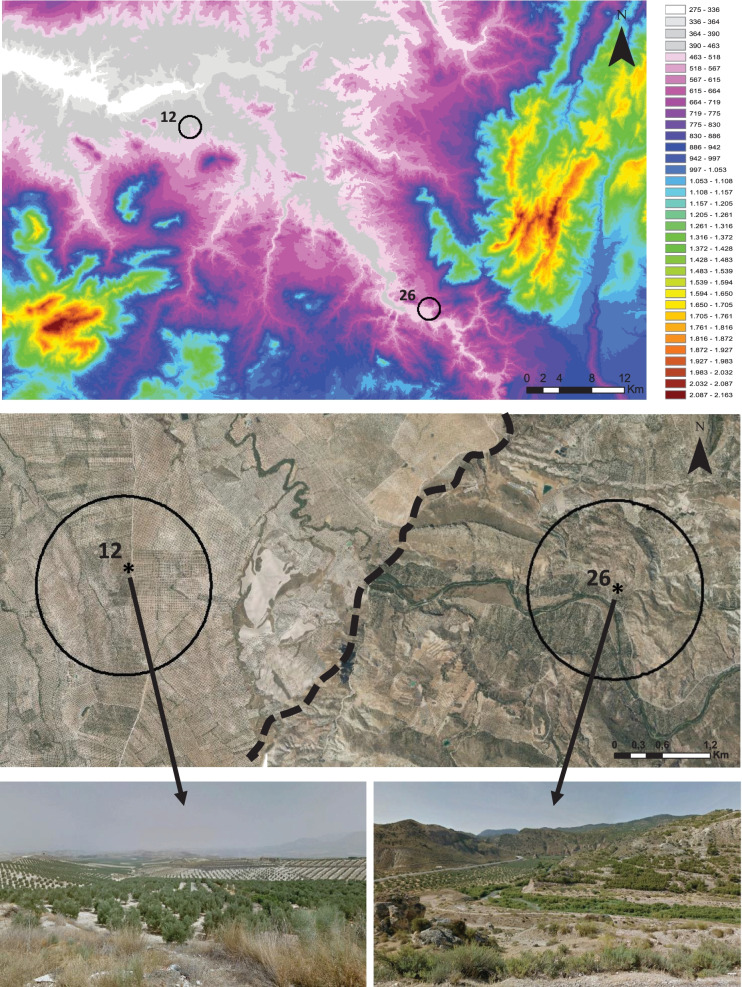
Altitude map of the study area and photographs of two representative landscape types. Number 12 is an example of a simple landscape dominated by olive groves and number 26 is an example of a complex landscape with different land uses around the olive groves

The study area, covering approximately 120,000 ha, was in the province of Jaén (Fig. 1), host to the largest cultivated area of olive groves in Spain (590,000 ha) (ESYRCE-MAPAMA, 2020). According to the Alert and Phytosanitary Information Network (RAIF) of Andalusia classification, the studied area includes part of four agroclimatic zones: *Loma Alta*, *Loma Baja*, *Mágina Norte* and *Sierra de Cazorla*. The soil is mainly classified as loam or clay loam and the climate is Mediterranean, with an average annual temperature of 17.3 °C and precipitation of 624 mm. The average altitude in the study area is 663 m a.s.l., varying between 345 and 1038 m a.s.l. (Fig. 2).

### Data collection B. oleae and P. oleae

Data on the populations of *B. oleae* and *P. oleae* were provided by the RAIF (https://www.juntadeandalucia.es/agriculturapescaydesarrollorural/raif). This network gathers data on population densities of different phytophagous insects and their levels of damage, as well as information on diseases affecting crops in Andalusia. These data came from 39 sampling points (SP), randomly selected and separated by at least 4 km (Fig. 1), which correspond to Biological Control Stations (BCS) where RAIF carried out weekly abundance data collection of *P. oleae* and *B. oleae*, according to the indications of the “Guía de Gestión Integral de Plagas del Olivar” (MAPAMA, 2014). *B. oleae* were present in 37 SP and *P. oleae* in 30. Average size of olive orchards was 16.06 ± 8,27 ha. In the case of *B. oleae*, three traps per BCS of yellow adhesive chromotropic type baited with sex pheromones were used. Their distribution in the BCS was random, respecting a minimum separation of 40 m between them. For *P. oleae*, two funnel traps were set, also containing sex pheromones as an attractant and again with a random distribution but respecting a minimum distance of 50 m between them.

Sampling of both *P. oleae* and *B. oleae* was carried out weekly during 2009, 2010 and 2011, in all cases throughout their flight period, i.e. during their last stage of development. These years were chosen because of their higher presence of both pests. These samplings were adapted to the phenology of each pest. *B. oleae* has two generations, whose flight curves occur from June to the end of July and from the end of August to the end of October, respectively. Therefore, samples were taken from July to the end of the first half of November, analysing data from a total of 54 dates per SP in the 3 years. In the case of *P. oleae*, three generations were analysed. Their flight periods occur from mid-April to the end of May, from the beginning of June to mid-July and from the end of September to the beginning of November respectively. Collections were made on a total of 35 dates per SP per year. For *P. oleae*, only data from 2009 were used because in 2010 and 2011, the pest did not occur in these olive groves. For both species, the variable analysed was (Capture) “Average number of adults captured per trap and per day”.

### Spatial analysis of the landscape

The spatial scale selected for each species was based on the dispersive capacity of each pest as reported by companies that sell traps for these pests, since no scientific studies were found to measure it in detail. Two circular areas (CA) were plotted for each pest using each SP as a centre, with radii of 500 m and 1000 m in the case of *P. oleae* and 1000 m and 1500 m for *B. oleae*. Spatial land use coverage data were taken from the Spanish Land Occupation Information System (SIOSE) to determine the composition and configuration of the landscape surrounding each SP. For this study, the minimum digitised size for a polygon was 0.5 ha. The original SIOSE land use classification was summarised and reclassified into eleven types, assigning a single land use to each patch determined by the land use that covered at least 50% of its area: artificial (A), deciduous broadleaved (BD), evergreen broadleaved (BE), coniferous (F), crops (C), *dehesas* (DH), olive grove (O), grassland (P), riverbank (RB), sparse vegetation (SV) and scrubland (M). The verification and updating of land uses were performed by comparing the SIOSE information with aerial photographs taken in 2011 provided by the National Aerial Orthophotography Plan (PNOA) and the information provided by the Geographic Information System of Agricultural Plots (SIGPAC).

ArcGis 10.2.1 software was used for the analysis of the mapped information. Specifically, for the Cost-Distance landscape index, two layers were used: (i) one in which the displacement resistance values assigned to each land use are collected, representing the cost or difficulty for a species to move through the different types of habitats and (ii) the source layer from which the accumulated resistance cost begins and its destination, which was the SP. A total of 18 hypotheses were formulated in which a resistance value was given to the different land uses (Table 1). These cost values ranged from 1 to 100 and considered the trends observed in the analysis of simple correlations between the percentages of use and the abundance of pests at each time of their phenological cycles (Annex 1), as well as previous scientific knowledge. The first hypotheses are simple, giving a minimum cost to one land use and to the rest the same maximum cost value. The hypotheses H1, H2, H5 and H8 were designed to determine the possible benefit of a given land use on the pest, where the lowest cost was assigned to that land use (1) and the highest cost was assigned to the rest of the land uses (100). The inverses of these hypotheses (H3, H6 and H9) were established to determine the opposite effect, i.e. a barrier effect of a given land use. Three hypotheses (H4, H7 and H10) gave a value of 1 to the three most extensive uses in our study area along with olive groves, and H11 gave a value of 1 to the least extensive uses, namely sparse vegetation, woodlands (deciduous brodleaved, evergreen broadleaved and coniferous), *dehesas* and grasslands. Two hypotheses were used to study the effect of natural land uses on pests, H12 (woodland uses) and H13 (*dehesas* and grasslands). Finally, five hypotheses (H14, H15, H16, H17 and H18) combined different values in the most similar uses based on ecological characteristics and the results obtained from the previous hypotheses.

**Table 1:** Resistance values assigned to the different land uses in each of the 18 scenarios tested. These values ranged from 1 to 100, according to their influence on the displacement of the pest in the olive landscape. Acronyms indicated by artificial (A), deciduous broadleaved (BD), evergreen broadleaved (BE), coniferous (F), crops (C), *dehesas* (DH), olive grove (O), grassland (P), riverbank (RB), sparse vegetation (SV) and scrubland (M)

Hypothesis	A	BE/BD	C	DH	F	M	O	P	RB	SV
H1	100	100	100	100	100	100	1	100	100	100
H2	100	100	100	100	100	1	100	100	100	100
H3	1	1	1	1	1	100	1	1	1	1
H4	100	100	100	100	100	1	1	100	100	100
H5	100	100	1	100	100	100	100	100	100	100
H6	1	1	100	1	1	1	1	1	1	1
H7	100	100	1	100	100	100	1	100	100	100
H8	100	100	100	100	100	100	100	100	1	100
H9	1	1	1	1	1	1	1	1	100	1
H10	100	100	100	100	100	100	1	100	1	100
H11	100	1	100	1	1	1	1	1	100	1
H12	100	1	100	1	1	1	100	100	100	100
H13	100	100	100	1	100	100	100	1	100	100
H14	100	50	10	50	50	10	1	10	10	10
H15	100	10	10	10	50	10	1	10	10	10
H16	100	10	50	50	1	50	50	50	10	50
H17	100	10	50	50	10	50	50	50	1	50
H18	100	20	100	10	20	50	10	50	1	100

### Statistical analysis

Linear mixed effects models (GLMM) were applied for each scale and generation, using the Capture data as the dependent variable, previously transformed by log (*x* + 1) to normalise the data. Sampling points were considered as random effects and repeated measures per week as fixed effects. The cost-distance of the 18 hypotheses and the altitude of the different sampling points were considered as covariates. Altitude was included due to its relationship with *B. oleae* populations (Kounatidis et al., 2008); however, in *P. oleae*, the effect was unknown.

Models were fitted using a restricted maximum likelihood estimation method. The best covariance structure for the repeated measures factor was Diagonal, which was selected according to the lowest value of the Akaike and Schwarz Bayesian information criteria fit statistics (Littell et al., 1998; Wang & Goonewardene, 2004). IBM SPSS Statistic 22.0 software was used to compute the statistical analyses.

## Results

### Relationship between landscape structure and pest abundance

**Fig. 3:**
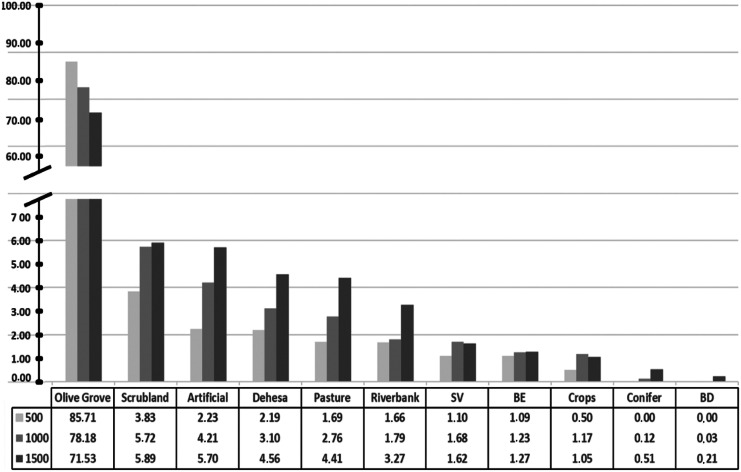
Percentage of surface area occupied by different land uses in the study area. This was quantified at three scale levels: 500, 1000 and 1500 m radius for each plot

Olive groves were the main land use in the study area, occupying an average percentage of 85.71% of the land area at the 500-m radius spatial scales, 78.18% at 1000 m and 71.53% at 1500 m. For the 1000 m radius CAs, the remaining land uses of scrubland, pasture and crops had a similar mean percentage occupancy of 4.21, 3.10 and 5.72%, respectively. The remaining land uses occupied less than 2% of the area (deciduous hardwoods 0.03%, sparse vegetation 1.68%, conifers 0.12%, *dehesas* 2.76%, artificial 1.79%, evergreen hardwoods 1.23% and riparian forests 1.17%). Percentages of occupancy at spatial scales from 500 to 1500 m radius followed a similar pattern (Fig. 3).

Significant correlations were found between the different types of land uses and pest abundances in some generations of their phenological cycle (Annex 1 and Annex 2). Negative correlations were found in the land uses RB, C and M up to the 1500 m scale for *B. oleae* and positive correlations with use C up to 1000 m in the case of *P. oleae*.

### Pest dispersal across the landscape

GLMMs indicating the relationship between pest abundance and the cost-distance variable in each of the hypotheses indicate the barrier or corridor effect of the different land uses at the scales analysed (Tables 2 and 3). Significant negative relationships indicate that the hypotheses are true and positive relationships indicate that they are false. Similar results were obtained with the two pairs of spatial scales used for the analysis of each species, indicating that all of them are suitable for the study of the movements of these pests. The effects that were found to be significant for each type of use are described below (Table 4).

**Table 2:** Results of the linear mixed effects models (GLMM) for 18 cost-distance hypotheses (H). The influence of the covariate altitude (Alt.) of the sampling points and the mean value of Cost-Distance of each hypothesis are shown. The dependent variable was the capture data for each pest. Results are shown for the two generations of *Bactrocera oleae* for the years 2009, 2010 and 2011 at two spatial scales: 1000 m and 1500 m around the sampling points

Scale	Parameters	Summer generation				Autumn generation			
		Altitude		Hypothesis		Altitude		Hypothesis	
		*t*	Significance	*t*	Significance	*t*	Significance	*t*	Significance
1000	Alt. + H1	8.06	0.000**	− 2.62	0.014**	4.77	0.000**	-	-
1500	Alt. + H1	6.35	0.000**	-	-	3.91	0.001**	-	-
1000	Alt. + H2	7.30	0.000**	-	-	4.53	0.000**	-	-
1500	Alt. + H2	6.92	0.000**	2.30	0.029**	4.09	0.000**	-	-
1000	Alt. + H3	7.43	0.000**	− 2.13	0.041**	4.40	0.000**	-	-
1500	Alt. + H3	6.75	0.000**	− 2.36	0.026**	3.98	0.001**	-	-
1000	Alt. + H4	7.73	0.000**	− 1.84	0.073*	5.16	0.000**	− 2.23	0.033**
1500	Alt. + H4	7.96	0.000**	− 3.12	0.004**	4.89	0.000**	− 2.28	0.031**
1000	Alt. + H5	7.33	0.000**	-	-	5.55	0.000**	3.09	0.004**
1500	Alt. + H5	6.41	0.000**	-	-	5.16	0.000**	3.08	0.005**
1000	Alt. + H6	6.99	0.000**	-	-	4.46	0.000**	− 2.99	0.005**
1500	Alt. + H6	6.15	0.000**	-	-	4.19	0.000**	− 4.11	0.000**
1000	Alt. + H7	8.04	0.000**	− 2.43	0.021**	4.55	0.000**	-	-
1500	Alt. + H7	8.02	0.000**	− 3.20	0.004**	4.40	0.000**	-	-
1000	Alt. + H8	7.35	0.000**	-	-	3.75	0.001**	-	-
1500	Alt. + H8	5.80	0.000**	-	-	2.29	0.030**	1.80	0.083*
1000	Alt. + H9	6.40	0.000**	-	-	3.29	0.003**	-	-
1500	Alt. + H9	5.80	0.000**	-	-	3.05	0.005**	− 2.43	0.021**
1000	Alt. + H10	8.59	0.000**	− 2.75	0.010**	4.77	0.000**	-	-
1500	Alt. + H10	8.19	0.000**	− 3.37	0.002**	4.46	0.000**	-	-
1000	Alt. + H11	7.00	0.000**	-	-	4.48	0.000**	− 3.08	0.004**
1500	Alt. + H11	6.11	0.000**	-	-	4.09	0.000**	− 4.27	0.000**
1000	Alt. + H12	8.00	0.000**	2.40	0.023**	4.85	0.000**	1.18	0.079*
1500	Alt. + H12	6.98	0.000**	1.89	0.069*	4.24	0.000**	-	-
1000	Alt. + H13	8.53	0.000**	3.01	0.005**	4.37	0.000**	-	-
1500	Alt. + H13	8.81	0.000**	4.07	0.000**	3.92	0.001**	-	-
1000	Alt. + H14	8.80	0.000**	− 2.14	0.039**	5.88	0.000**	− 2.03	0.050**
1500	Alt. + H14	6.19	0.000**	− 1.73	0.095*	4.29	0.000**	− 1.86	0.075*
1000	Alt. + H15	9.15	0.000**	− 2.89	0.007**	5.60	0.000**	− 1.79	0.082*
1500	Alt. + H15	6.76	0.000**	− 2.77	0.010**	4.03	0.001**	-	-
1000	Alt. + H16	8.12	0.000**	-	-	4.43	0.000**	-	-
1500	Alt. + H16	6.06	0.000**	-	-	3.17	0.004**	-	-
1000	Alt. + H17	8.06	0.000**	-	-	4.06	0.000**	-	-
1500	Alt. + H17	6.07	0.000**	-	-	2.92	0.007**	-	-
1000	Alt. + H18	8.86	0.000**	− 2.15	0.039**	5.11	0.000**	-	-
1500	Alt. + H18	6,79	0,000**	− 2.65	0.014**	3.66	0.001**	-	-

*p<0.10; **p<0.05

**Table 3:** Results of the linear mixed effects models (GLMM) for the 18 hypotheses (H), where the influence of the covariate altitude (Alt.) of the sampling points and the mean value of Cost-Distance of each hypothesis by plot are shown. The dependent variable was the capture data for each pest. Results are shown for the three generations of *Prays oleae* for the year 2009 at two spatial scales: 500 m and 1000 m around the sampling point

		First generation				Second generation				Third generation			
		Altitude		Hypothesis		Altitude		Hypothesis		Altitude		Hypothesis	
Scale	Parameters	*t*	Sig	*t*	Sig	*t*	Sig	*t*	Sig	*t*	Sig	*t*	Sig
500 1000	Alt. + H1	-	-	− 2.69	0.081*	-	-	-	-	-	-	-	-
	Alt. + H1	− 2.69	0,013**	− 2.59	0.017**	-	-	-	-	-	-	-	-
500 1000	Alt. + H2	-	-	-	-	-	-	-	-	-	-	-	-
	Alt. + H2	− 2.86	0.008**	-	-	-	-	-	-	-	-	-	-
500 1000	Alt. + H3	-	-	− 2.23	0.049**	-	-	-	-	-	-	-	-
	Alt. + H3	-	-	− 3.15	0.004**	-	-	-	-	-	-	-	-
500 1000	Alt. + H4	-	-	-	-	-	-	-	-	-	-	− 2.14	0.036**
	Alt. + H4	− 2.09	0.047**	-	-	-	-	-	-	-	-	-	-
500 1000	Alt. + H5	-	-	-	-	-	-	-	-	-	-	− 3.19	0.003**
	Alt. + H5	− 2.52	0.018**	-	-	-	-	-	-	-	-	− 2.46	0.025**
500 1000	Alt. + H6	-	-	-	-	-	-	-	-	-	-	3.07	0.004**
	Alt. + H6	− 2.50	0.019**	-	-	-	-	-	-	-	-	-	-
500 1000	Alt. + H7	-	-	− 1.86	0.093*	-	-	-	-	-	-	-	-
	Alt. + H7	− 2.79	0.010**	− 2.43	0.023**	-	-	-	-	-	-	-	-
500 1000	Alt. + H8	-	-	-	-	-	-	-	-	-	-	-	-
	Alt. + H8	− 2.37	0.025**	-	-	-	-	-	-	-	-	1.76	0.093*
500 1000	Alt. + H9	-	-	-	-	-	-	-	-	-	-	-	-
	Alt. + H9	− 2.51	0.019**	-	-	-	-	-	-	-	-	− 1.75	0.091*
500 1000	Alt. + H10	-	-	− 1.93	0.083*	-	-	-	-	-	-	-	-
	Alt. + H10	− 2.56	0.017**	− 0.58	0.017**	-	-	-	-	-	-	-	-
500 1000	Alt. + H11	− 1.90	0.081*	− 2.19	0.052*	-	-	-	-	-	-	-	-
	Alt. + H11	− 2.74	0.011**	-	-	-	-	-	-	-	-	2.01	0.062*
500 1000	Alt. + H12	-	-	-	-	-	-	-	-	-	-	1.79	0.078**
	Alt. + H12	− 2.61	0.015**	-	-	-	-	-	-	-	-	-	-
500 1000	Alt. + H13	-	-	-	-	-	-	-	-	-	-	-	-
	Alt. + H13	− 2.88	0.008**	2.81	0.010**	-	-	-	-	-	-	-	-
500 1000	Alt. + H14	-	-	− 3.45	0.002**	2.27	0.027**	-	-	3.26	0.003**	− 2.27	0.029**
	Alt. + H14	-	-	− 2.46	0.021**	2.23	0.030**	-	-	3.43	0.002**	− 2.48	0.018**
500 1000	Alt. + H15	− 1.85	0.077*	− 4.45	0.000**	2.15	0.036**	-	-	2.88	0.007**	-	-
	Alt. + H15	-	-	− 3.91	0.001**	-	-	-	-	2.87	0.007**	− 1.92	0.063*
500 1000	Alt. + H16	-	-	-	-	2.01	0.040**	-	-	2.58	0.015**	-	-
	Alt. + H16	-	-	-	-	-	-	-	-	-	-	-	-
500 1000	Alt. + H17	-	-	-	-	1.96	0.055*	-	-	2.50	0.018**	-	-
	Alt. + H17	-	-	-	-	-	-	-	-	-	-	-	-
500 1000	Alt. + H18	-	-	− 3.49	0.002**	2.23	0.030**	-	-	2.83	0.008**	-	-
	Alt. + H18	-	-	− 3.29	0.003**	2.23	0.030**	-	-	2.85	0.008**	-	-

*p<0.10; **p<0.05

**Table 4:** Interpretation of the effect of each land use on *B. oleae* and *P. oleae* detected in the hypotheses that proved true in the GLMMs. 1G, 2G and 3G identify the pest generation on which the effect was detected. N.k. indicates not known. Hnumber identifies hypothesis

Land use	Hypothesis	*B. oleae*	*P. oleae*
Olive grove	H1 H7 (olive and crops) H10 (olive and riverbank)	Corridor 1G Corridor 1G Corridor 1G	Corridor 1G Corridor 1G Corridor 1G
Scrubland	H2 H3 H4 (olive and scrubland)	n.k Barrier 1G Corridor 1G and 2G	Corridor 3G Barrier 1G Corridor 3G
Crop	H5 H6	n.k Barrier 2G	Corridor 3G n.k
Riverbank	H9 H10 H18	Barrier 2G Corridor 1G Corridor 1G	Barrier 3G Corridor 1G Corridor 1G
Woodland uses	H14 H15 (only conifer)	Barrier 1G and 2G Barrier 1G and 2G	Barrier 1G and 3G Barrier 1G and 3G
Dehesas and grasslands	H13	Barrier 1G	Barrier 1G

Olive groves showed a corridor effect in the first generation of both pests (H1). In addition, they showed the same effect in combination with other less extensive land uses, such as crops and riverbanks in the same generations (H7 and H10). Shrublands showed a barrier effect in the first generation of both pests (H3), but in combination with olive groves, they showed a corridor effect in the two generations of *B. oleae* and in the third generation of *P. oleae* (H4). H2 was not significant for *B. oleae*, but was significant for *P. oleae* in its third generation, which showed a corridor effect. Crops showed a corridor effect only in the third generation of *P. oleae* (H5) and a barrier effect in the second generation of *B. oleae* (H6). Riverbanks showed a barrier effect in the last generation of both pests (H9) and a corridor effect in the first generation (H10). Woodlands land uses showed barrier effects in all generations of both pests, both grouping broadleaves with conifers (H14) and conifers alone (H15). *Dehesas* and grassland also showed barrier effects for both pests in the first generation (H13).

In hypothesis H11, minimum values were assigned to woodland, *dehesas* and grasslands and maximum values to crops and riverbanks. The GLMMs were weakly significant with a negative relationship in the first generation of *P. oleae* and last generation of *B.oleae* and a positive relationship in the last generation of *P. oleae*. Hypotheses H16 and H17 present conifers and riverbanks as favourable pest habitats (cost 1), better than olive groves (cost 50) and other land uses. None was significant for either pest. Hypothesis H18 presents only riverbanks as pest corridors (cost 1), better than olive groves (cost 10) and other uses with higher costs. The models indicated that for the first generations of both pests, riverbanks may serve as summer corridors.

## Discussion

### Effectiveness of the methodology for the detection of pest dispersal capacity in agricultural landscapes

Spatial analysis, based on the cost that land uses have for pest movement in an agricultural landscape, has been effective in detecting the barrier or corridor effects of surrounding land uses. This study demonstrates that the cost-distance tool can aid researchers in the study of pest control and can support decision-makers and farmers in their transition to digitised agriculture. The application of simplified hypotheses to detect the spatial effects of land use connectivity was also employed by Perović et al. (2010) for detecting the effectiveness of biological pest control of cotton pests. However, this approach had not been used for pest displacement. In our case, the direction of displacement is from the edge of the circular area analysed to the centre, which is our sampling point. Therefore, a negative relationship between the recorded pest abundance and the hypothetical cost of traversing a land use indicates support for the hypothesis. On the contrary, a positive relationship indicates the hypothesis may be false.

In addition, permeability for the pest species studied through the cost-distance tool provides a functional input that allows the inference of spatial connectivity as well as functionality. In general, the analysis of landscape connectivity is usually focused on wildlife conservation detecting links between specific natural land uses and land covers with respect to a particular species (Beier et al., 2011). The results of our study focusing on permeability show, in addition to its implications for the physical structure of the landscape and the dispersal of organisms, the potential of that landscape in supporting the continuous flow of natural processes, that is, ecological flows (Anderson & Clark, 2012).

#### Influence of different land uses on the movement of B. oleae and P. oleae

The dispersal ability of a taxon across the landscape is marked both by its physiology and by the influence exerted by different land uses and the geographic particularity of the terrain (Henle et al., 2004; Reynolds et al., 2018; Tscharntke et al., 2012). Thus, the same landscape element can trigger different responses in the dispersive capacity of different taxa. A particular land use may simultaneously act as a corridor, food source or ecological barrier (Chakraborty et al., 2017; Gontijo, 2019).

Olive groves account for most of the land use in the study area. Hypotheses were developed assigning the minimum resistance for pest movement to this land use, either alone (H1) or in combination with one other land use (H4, H7 and H10 with scrubland, crops and riverbank, respectively). Generally speaking (see Table 4), the results show that both pests use the olive grove as a habitat spending more of each phenological cycle there (refuge), such that pest abundance increases as the degree of connection and extent of olive groves increases (Ortega et al., 2018). Some recent studies support the greater efficacy of biological control in more complex landscape contexts (Dainese et al., 2017; Pérez-Álvarez et al., 2019). However, there remains some inconsistency in the direct benefits of the diversity of surrounding non-agricultural land uses on this ecosystem service (Holland et al., 2017; Karp et al., 2018). Some experimental work supports the “intermediate landscape complexity hypothesis”. This hypothesis predicts that local conservation management for biological control will be most effective in moderately simple agricultural landscapes, and less effective in very simple or very complex landscapes. In the first case, this is due to a lack of responsiveness and in the second case, to the fact that the response potential could be saturated (Jonsson et al., 2015) or because there may be intra-gremial predation or behavioural interference among natural enemies (Finke & Denno, 2005). In this framework of controversy, our results provide an interesting perspective as they allow management considerations that are directly linked to the biology of the pests themselves. In the specific case of these hypotheses, it is evident that the tendency to homogenise olive landscapes would lead to the increase in *B. oleae* and *P. oleae* pests by facilitating their dispersal. According to Paredes et al., (2013), the best option to improve the effectiveness of biological control in this type of simplified and homogeneous landscape could be the implementation of grass strips or scrubs and hedges.

For the *s*crubland vegetation cover, H2 and H3 were designed, based on previous studies (Boccaccio & Petacchi, 2009; Ortega et al., 2018; Villa et al., 2020) that indicated the negative relationship of scrublands and the densities of both pests. This type of natural or semi-natural vegetation is favourable for the 3rd generation of *P. oleae* and acts as a barrier for the first generation of both pests, perhaps due to the presence of natural predators (see Table 4). Some of these pests belong to the orders Heteroptera (Pentatomidae), Demaptera (Forficulidae), Coleoptera (Carabidae) and Hymenoptera (Formicidae). This was demonstrated by Rejili et al. (2016) who found sequences of cytochrome oxidase subunit I (COI) gene of *B. oleae* and *P. oleae* in the digestive tract of some of these arthropods. Therefore, in the two pests considered, scrubs seem to exert a barrier effect in their displacement in the first generation and a corridor effect in the third generation of *P. oleae*, when the predators may be undergoing a population decline. The barrier effect is likely due to the biological control exerted by the presence of natural enemies that would reduce the populations of these pests or to the absence of attractive food in these natural patches. However, the corridor effect in the third generation of *P. oleae* may be due to the presence of food plant species (Villa et al., 2020).

The possible relevance of the presence of natural or semi-natural vegetation covers near olive groves or other woody crops to improve conservation biological control and other ecosystem services has been studied by several authors (Alves et al., 2021; Paredes et al., 2019; Villa et al., 2020; Wu et al., 2019). In olive groves, Paredes et al., (2019) suggest that the maintenance of surrounding natural and semi-natural habitats may be a promising practice to promote the presence of natural enemies to control *P. oleae* infestations. Particularly, the importance of scrublands as providers of ecosystem services has been shown in different case studies. For example, *B. oleae* predation in olive groves was associated with the area of scrubland in autumn (Ortega et al., 2018). However, it is important to also consider flowering plants with potential benefits to pests. In this regard, Wu et al. (2019) showed that the availability of local floral resources and nearby natural scrublands seemed especially important for enhancing wild bee abundance and their potential services in apple crops.

H5, H6 and H7 were designed to analyse the isolated effect exerted by crops, understood as areas surrounding the olive grove where farms cultivating cereals and small irrigated areas prevail. The predominant barrier effect on *B. oleae* was corroborated and could be due to the physical–chemical alterations derived from agricultural activities, specifically the use of phytosanitary products and the tilling of these cultivation areas in the summer-fall season, which physically removes the plant cover (Thorbek & Bilde, 2004; Tscharntke et al., 2016). However, only for the third generation of *P. oleae* was a synergistic effect observed between the olive grove and crop patches, where these patches favour the spread of the pest. Despite having a strong agricultural tillage character, some research has found that in late summer, this pest could find in these patches certain plant species—the so-called weeds—that could serve as a complementary food source (Nave et al., 2021). Furthermore, as observed by Paredes et al. (2013), these land uses are characterised by the presence of bare soils for long periods of time as well as the absence of surrounding natural vegetation, which significantly decreases the biological control exerted by the natural enemies of these pests.

For the riverbank, with H9, the barrier effect was observed in the last generation of both pests. However, with H10, a corridor effect was observed in the first generations of both pests that were corroborated by H18. This means that riverbanks associated with watercourses exert a summer corridor effect and a barrier effect at the end of the biological cycle probably due to the influence of temperature. In olive groves in Morocco, Mansour et al. (2017) found that coastal areas with mild temperatures and significant humidity had high densities of adult populations of *P. oleae*, whereas in inland areas with a warmer and less humid climate, emergences are late, and densities are lower. Thus far, wetlands or areas with nearby water availability have not been shown to be refuges for natural enemies of agricultural pests.

Hypotheses H14 and H15 confirm that woodlands show a barrier effect on the two generations of *B. oleae* and the first and third generations of *P. oleae*. Natural enemies of *P.oleae*, for example the *Anthocoridae Anthocoris nemoralis (Fabricius*) that consume eggs of the second generation of *P. oleae*, were favoured by the occurrence of large woody patches surrounding the olive groves (Paredes et al., 2013). Similar results were obtained by Boccaccio and Petacci (2009), who showed that the parasitism rate of the olive fly was favoured by the occurrence of woodlands (forest and scrub). Natural vegetation patches in large agricultural areas seem to be one of the major reservoirs and main habitats for natural predators of pests (Galloway et al., 2021; Paredes et al., 2019).

H13 focused on the role of grasslands and *dehesas* as corridors, the former occupying a larger area in our study area. Both share ecological characteristics with each other and with the olive grove (López-Fernández, 2011). The herbaceous tapestry is common in organic olive orchards, as are scattered trees in holm oak pastures with a density and tree size similar to the olive groves. The models with this hypothesis were false or non-significant. Thus, these land uses could be considered barriers in the first generation, with unknown implications on the displacement of these pests in later generations. Recently, Álvarez et al. (2021) highlighted the effect of ground cover in organic olive orchards increasing interactions with natural enemies against *P. oleae*, promoting effective egg predation. Hence, the role of these land uses as ecological barriers seems to be well-established.

#### Effect of spatial scale

According to some trap companies, the imago dispersal capacity of *P. oleae*, in each of the three generations, does not exceed 1000 m from the point of imago development. Therefore, we used 500 and 1000 m radii as the action ranges for *P. oleae*, since we found no studies that demonstrated other dispersion capacity distances. However, the results obtained in the GLMMs are not conclusive and only a few patterns can be found that vary based on the generation studied. In the first generation, it seems that the landscape has a more direct influence at the 1000 m radius scale, which agrees with previous research showing stronger landscape effects at larger scales (750 and 1000 m), compared to smaller scales (500 and 600 m) (Costa et al., 2020; Villa et al., 2020). In the second and third generations, few significant patterns are found.

In the case of *B. oleae*, the literature indicates a dispersive ability, where the movement of some individuals has been monitored from 150 m (Rempoulakis1 & Nestel, 2011) up to 400 m (Fletcher & Economopoulos, 1976). However, Ortega and Pascual (2014) found an important spatial influence of the landscape on population density between 1000 and 1500 m radius. Therefore, we selected a range of action between 1000 and 1500 m radius. We found for both generations a greater effect of the landscape, and thus a greater number of interactions with a higher significance value, at the 1000 m radius.

#### Effect of altitude on the dispersal behaviour of these pests

Altitude has a direct relationship on the distribution of *B. oleae*, with higher fly population densities as altitude increases (Kounatidis et al., 2008). This behaviour is likely driven by the need for adults to find refuge from the high temperatures that occur in Mediterranean environments during the summer and early autumn months at higher altitudes. However, in the summer generation, higher values of significance were found (Table 2). This is related to the fact that during this period, peaks of up to 40 °C are reached with a low relative humidity that can reach values of up to 34% in contrast to the autumn months where the average temperature is 13.42 °C with a relative humidity of 63.75% according to the State Meteorological Agency (AEMET). Wang et al. (2009) indicated that high summer temperatures affect the survival and reproduction of olive fruit flies.

In the case of *P. oleae*, the effect is heterogeneous, since it has been observed that the influence of altitude depends on the generation studied. In the case of the first generation, this lepidopteran prefers lower altitudes where temperatures are higher during the spring months. On the contrary, in the second and third generations, the sign of the relationship is inverted.

### Usefulness of the cost-distance tool in the context of smart agriculture

Agriculture is moving towards the use of digital tools driven by technological innovation. New concepts of traceability, quality and control are gaining ground, linked to society’s greater sensitivity to environmental problems and awareness of the need for better management of available resources. The agricultural sector is currently in the early stages of what is known as agriculture 4.0. This refers to agricultural interventions that leverage accurate and timely analyses of data and information collected and transmitted through advanced tools and technologies. This type of smart farming is based on tools and strategies that allow a synergistic use of a range of digital technologies for the automatic collection, integration and analysis of data from the field, from sensors or from third parties (Bacco et al., 2019). From these technologies, farmers obtain increasingly accurate support in their decision-making process, both in terms of their own activities and their relationship to other parts of the supply chain. Ultimately, the goal of agricultural digitisation is to increase profitability and economic, environmental and social sustainability of agricultural processes. In the specific case of the olive groves studied here, the most relevant result provided by the cost-distance model applied at different scales for the two pests indicates the effectiveness of maintaining or promoting natural or semi-natural land uses (i.e. heterogenising the landscape) to improve the control of both pests. The simplicity, precision and speed of the software required for the tool favour its application and would imply little investment by farmers as the information would come from managers, in this case the Integrated Treatment Groups in Agriculture (ATRIAS). In the same line, this tool is useful in land-use planning that maximises spatial resilience to pests and its results are corroborated by the positive relationship between olive landscape heterogeneity and spatial resilience (Rescia & Ortega, 2018).

Indeed, the use of Information and Communication Technology (ICT) in the field of agriculture is a first step in the intense digital transformation that is demanded by current global challenges, such as a growing world population accompanied by an increasing demand for food and climate change (Vermeulen et al., 2012). However, recurrent constraints are that the results obtained in experimental studies on technological benefits in agriculture reach the farmers through the managers, and that farmers are willing to follow the recommendations derived from these results. A challenge in the short term is to facilitate the mechanisms of transmission of experimental results to managers and from managers to farmers. Other authors, such as Rescia et al. (2017), proposed an environmental payment mechanism for olive farmers and discussed in their study the importance of applying this mechanism by facilitating transmission channels between managers, decision-makers and farmers.

## Conclusions

The study of landscape connectivity using the Cost-Distance tool allows us to determine the ecological role of each land use in the olive landscape, in relation to the two main olive pests. While some natural land uses such as woodlands impair the movement of these pests, others such as riverbanks or scrublands act as a corridor or barrier, depending on the phenological cycle of each pest. This could indicate a greater dispersive capacity of these pests during the spring months, which has already been observed in the case of *P. oleae* (Villa et al., 2020), and in *B. oleae* (Ortega et al., 2021). This seems obvious as in spring and early summer there are no olives in the trees. For this reason, promoting those land uses that are most harmful to pests (that exert a barrier effect) such as woodlands, *dehesas*, grasslands and scrublands will reduce the dispersion ability and the population densities of these pests by means of natural enemies living in these environments, hence mitigating the ecological impact of these pests on the olive-growing ecosystem. Nonetheless, the influence of other environmental variables such as altitude or the spatial scale at which these land uses exert greater pressure, as well as the true dispersive ability of both pests, should be studied in depth. The conclusions of this work have been obtained by means of indirect effects. However, its applicability in spatial planning and digitisation of agriculture seems certain due to the consistence of the results obtained. Nevertheless, more detailed studies on population densities of these pests and their natural enemies in other landscapes are needed to determine the direct effects.

## Data Availability

The datasets generated during and/or analysed during the current study are available from the corresponding author on reasonable request.

## Annex 1

Pearson correlation analysis between abundance of *Prays oleae* (with a logarithm transformation) and percentage of different land uses. *N* is number of sampling points. Acronyms indicated by artificial (A), deciduous broadleaved (BD), evergreen broadleaved (BE), coniferous (F), crops (C), dehesas (DH), olive grove (O), grassland (P), riverbank (RB), sparse vegetation (SV) and scrub (M).

**Table:**

Year	Scale	**Pearson correlation**														
		Generation		%O	%M	%C	%P	%DH	%RB	%A	%SV	%BE	%BD	%F	% (DH_P)	Forest
2009	500	1er	*r*	-	-	-	-	-	-	-	-	-	-	-	-	-
			Significance *N*	-	-	-	-	-	-	-	-	-	-	-	-	-
2009	500	2nd	*r*	-	-	-	-	-	-	-	-	-	-	-	-	-
			Significance	-	-	-	-	-	-	-	-	-	-	-	-	-
2009	500	3rd	*r*	-	-	0.472	-	-	-	-	-	-	-	-	-	-
			Significance *N*	-	-	0.088 (14)	-	-	-	-	-	-	-	-	-	-
2009	1000	1er	*r*	-	-	-	-	-	-	-	-	-	-	-	-	-
			Significance *N*	-	-	-	-	-	-	-	-	-	-	-	-	-
2009	1000	2nd	*r*	-	-	-	-	-	-	-	-	-	-	-	-	-
			Significance *N*	-	-	-	-	-	-	-	-	-	-	-	-	-
2009	1000	3rd	*r*	-	-	0.508	-	-	-	-	-	− 0.533^*^	-	-	-	-
			Significance *N*	-	-	0.064 (14)	-	-	-	-	-	0.050 (14)	-	-	-	-

**p*<0.10; ***p*<0.05

## Annex 2

Pearson correlation analysis between abundance of *Bactrocera oleae* (with a logarithm transformation) and percentage of different land uses. *N* is number of sampling points. Acronyms indicated by artificial (A), deciduous broadleaved (BD), evergreen broadleaved (BE), coniferous (F), crops (C), dehesas (DH), olive grove (O), grassland (P), riverbank (RB), sparse vegetation (SV) and scrub (M).

**Table:**

Year	Scale	**Pearson correlation**													
		Generation		%O	%M	%C	%P	%DH	%RB	%A	%SV	%BE	%BD	%DH_P	Forest
2009	1000	Summer	*r*	-	-	-	-	-	− 0.430^*^	-	-	-	-	-	-
			Significance *N*	-	-	-	-	-	0.011 (34)	-	-	-	-	-	-
2009	1000	Autumn	*r*	-	-	− 0.392^*^	-	-	-	-	-	-	-	-	-
			Significance *N*	-	-	0.022 (34)	-	-	-	-	-	-	-	-	-
2009	1500	Summer	*r*	-	− 0.326	-	-	-	− 0.482^**^	-	-	-	-	-	-
			Significance *N*	-	0.079 (30)	-	-	-	0.007 (30)	-	-	-	-	-	-
2009	1500	Autumn	*r*	-	-	− 0.491^**^	-	-	− 0.472^**^	-	-	-	-	-	-
			Significance *N*	-	-	0.006 (30)	-	-	0.009 (30)	-	-	-	-	-	-
2010	1000	Summer	*r*	-	-	-	-	-	− 0.310	-	-	-	-	-	-
			Significance *N*	-	-	-	-	-	0.096 (30)	-	-	-	-	-	-
2010	1000	Autumn	*r*	-	-	-	-	-	-	-	-	-	-	-	-
			Significance *N*	-	-	-	-	-	-	-	-	-	-	-	-
2010	1500	Summer	*r*	-	− 0.373	-	-	-	-	-	-	-	-	-	-
			Significance *N*	-	0.061 (26)	-	-	-	-	-	-	-	-	-	-
2010	1500	Autumn	*r*	-	-	-	-	-	-	-	-	-	-	-	-
			Significance *N*	-	-	-	-	-	-	-	-	-	-	-	-
2011	1000	Summer	*r*	-	-	-	-	-	− 0.426^*^	-	-	-	-	-	-
			Significance *N*	-	-	-	-	-	0.030 (26)	-	-	-	-	-	-
2011	1000	Autumn	*r*	-	-	− 0.461^*^	-	-	-	-	-	-	-	-	-
			Significance *N*	-	-	0.018 (26)	-	-	-	-	-	-	-	-	-
2011	1500	Summer	*r*	-	-	-	-	-	− 0.488^*^	-	-	-	-	-	-
			Significance *N*	-	-	-	-	-	0.021 (22)	-	-	-	-	-	-
2011	1500	Autumn	*r*	-	-	− 0.509^*^	-	-	− 0.477^*^	-	-	-	-	-	-
			Significance *N*	-	-	0.016 (22)	-	-	0.025 (22)	-	-	-	-	-	-

**p*<0.10; ***p*<0.05

## References

[CR1] Adriaensen F, Chardon JP, De Blust G, Swinnen E, Villalba S, Gulinck H, Matthysen E (2003). The application of ‘least-cost’modelling as a functional landscape model. Landscape and Urban Planning.

[CR2] Álvarez HA, Jiménez-Muñoz R, Morente M, Campos M, Ruano F (2021). Ground cover presence in organic olive orchards affects the interaction of natural enemies against *Prays oleae*, promoting an effective egg predation. Agriculture, Ecosystems & Environment.

[CR3] Alves JF, Mendes S, Alves da Silva A, Sousa JP, Paredes D (2021). Land-use effect on olive groves pest Prays oleae and on its potential biocontrol agent Chrysoperla carnea. Insects.

[CR4] Anderson M, Clark M (2012). Modeling landscape permeability: A description of two methods to model landscape permeability.

[CR5] ArcGIS Desktop. (2020). ArcMap. Herramienta Coste-Distancia. Retrieved December 8, 2018, from https://desktop.arcgis.com/es/arcmap/10.3/tools/spatial-analyst-toolbox/cost-distance.htm

[CR6] Bacco M, Barsocchi P, Ferro E, Gotta A, Ruggeri M (2019). The digitisation of agriculture: A survey of research activities on smart farming. Array.

[CR7] Beier P, Majka DR, Spencer WD (2008). Forks in the road: Choices in procedures for designing wildland linkages. Conservation Biology.

[CR8] Beier P, Majka DR, Newell SL (2009). Uncertainty analysis of least-cost modeling for designing wildlife linkages. Ecological Applications.

[CR9] Beier P, Spencer W, Baldwin RF, McRAE BH (2011). Toward best practices for developing regional connectivity maps. Conservation Biology.

[CR10] Bianchi FJ, Booij CJH, Tscharntke T (2006). Sustainable pest regulation in agricultural landscapes: A review on landscape composition, biodiversity and natural pest control. Proceedings of the Royal Society b: Biological Sciences.

[CR11] Boccaccio L, Petacchi R (2009). Landscape effects on the complex of *Bactrocera oleae* parasitoids and implications for conservation biological control. BioControl.

[CR12] Cardinale BJ, Duffy JE, Gonzalez A, Hooper DU, Perrings C, Venail P, Narwani A, Mace GM, Tilman D, Wardle DA, Kinzig AP, Daily GC, Loreau M, Grace JB, Larigauderie A, Srivastava D, Naeem S (2012). Biodiversity loss and its impact on humanity. Nature.

[CR13] Chakraborty S, Tiwari PK, Sasmal SK, Biswas S, Bhattacharya S, Chattopadhyay J (2017). Interactive effects of prey refuge and additional food for predator in a diffusive predator-prey system. Applied Mathematical Modelling.

[CR14] Conolly J, Lake M (2006). Geographical information systems in archaeology.

[CR15] Costa A, Silva B, Jimenez-Navarro G, Barreiro S, Melguizo-Ruiz N, Rodríguez-Pérez J, Vasconcelos S, Beja P, Moreira F, Herrera JM (2020). Structural simplification compromises the potential of common insectivorous bats to provide biocontrol services against the major olive pest *Prays oleae*. Agriculture, Ecosystems & Environment.

[CR16] Coulon A, Cosson JF, Angibault JM, Cargnelutti B, Galan M, Morellet N, Petit E, Aulagnier S, Hewison AJM (2004). Landscape connectivity influences gene flow in a roe deer population inhabiting a fragmented landscape: An individual–based approach. Molecular Ecology.

[CR17] Dainese M, Schneider G, Krauss J, Steffan-Dewenter I (2017). Complementarity among natural enemies enhances pest suppression. Scientific Reports.

[CR18] Driezen K, Adriaensen F, Rondinini C, Doncaster CP, Matthysen E (2007). Evaluating least-cost model predictions with empirical dispersal data: A case-study using radiotracking data of hedgehogs (Erinaceus europaeus). Ecological Modelling.

[CR19] ESYRCE (Encuesta sobre Superficies y Rendimientos de Cultivos) -MAPAMA (Ministerio de Agricultura y Pesca, Alimentación y Medio Ambiente). (2020). Retrieved 2020 from https://www.mapa.gob.es/es/estadistica/temas/estadisticas-agrarias/boletin2020_tcm30-564330.pdf

[CR20] EU (European Union). (2018). DG Agriculture and Rural Development, Unit Farm Economics: Production, yields and productivity. Retrieved January 20, 2022 from https://ec.europa.eu/info/sites/default/files/food-farming-fisheries/farming/documents/production-yields-productivity_en.pdf

[CR21] Finke DL, Denno RF (2005). Predator diversity and the functioning of ecosystems: The role of intraguild predation in dampening trophic cascades. Ecology Letters.

[CR22] Fletcher BE, Economopoulos AP (1976). Dispersal of normal and irradiated laboratory strains and wild strains of olive fly *Dacus oleae* in an olive grove. Entomologia Experimentalis Et Applicata.

[CR23] Foley, J. A., DeFries, R., Asner, G. P., Barford, C., Bonan, G., Carpenter, S. R., Chapin, F. S., Coe, M. T., Daily, G. C., Gibbs, H. K., Helkowski, J. H., Holloway, T., Howard, E. A., Kucharik, C. J., Monfreda, C., Patz, J. A., Prentice, I. C., Ramankutty, N., & Snyder, P. K. (2005). Global consequences of land use. *Science*, 309(5734), 570–574. 10.1126/science.1111772 10.1126/science.1111772 16040698

[CR24] Galloway AD, Seymour CL, Gaigher R, Pryke JS (2021). Organic farming promotes arthropod predators, but this depends on neighbouring patches of natural vegetation. Agriculture, Ecosystems & Environment.

[CR25] Gontijo LM (2019). Engineering natural enemy shelters to enhance conservation biological control in field crops. Biological Control.

[CR26] Henle K, Davies KF, Kleyer M, Margules C, Settele J (2004). Predictors of species sensitivity to fragmentation. Biodiversity & Conservation.

[CR27] Holland JM, Douma JC, Crowley L, James L, Kor L, Stevenson DR, Smith BM (2017). Semi-natural habitats support biological control, pollination and soil conservation in Europe. A Review. Agronomy for Sustainable Development.

[CR28] Jonsson M, Straub CS, Didham RK, Buckley HL, Case BS, Hale RJ, Gratton C, Wratten SD (2015). Experimental evidence that the effectiveness of conservation biological control depends on landscape complexity. Journal of Applied Ecology.

[CR29] Karp DS, Chaplin-Kramer R, Meehan TD, Martin EA, DeClerck F, Grab H, Gratton C, Hunt L, Larsen EA, Martinez-Salinas A, O´Rourle, M. E.,  (2018). Crop pests and predators exhibit inconsistent responses to surrounding landscape composition. Proceedings of the National Academy of Sciences.

[CR30] Kounatidis I, Papadopoulos NT, Mavragani-Tsipidou P, Cohen Y, Tertivanidis K, Nomikou M, Nestel D (2008). Effect of elevation on spatio-temporal patterns of olive fly (*Bactrocera oleae*) populations in northern Greece. Journal of Applied Entomology.

[CR31] Littell RC, Henry PR, Ammerman CB (1998). Statistical analysis of repeated measures data using SAS procedures. Journal of Animal Science.

[CR32] López-Fernández, A. (2011). El olivar: Entre la dehesa y la erosión. Boletín de la Real Academia de Córdoba de Ciencias, Bellas Letras y Nobles Artes, ISSN 0034–060X, *90*(160), 331–340.

[CR33] Mansour AA, Ouanaimi F, Chemseddine M, Boumezzough A (2017). Study of the flight dynamics of *Prays oleae* (Lepidoptera: Yponomeutidae) using sexual trapping in olive orchards of Essaouira region, Morocco. Journal of Entomology and Zoology Studies.

[CR34] Mazzi D, Dorn S (2012). Movement of insect pests in agricultural landscapes. Annals of Applied Biology.

[CR35] Meehan TD, Werling BP, Landis DA, Gratton C (2011). Agricultural landscape simplification and insecticide use in the Midwestern United States. Proceedings of the National Academy of Sciences.

[CR36] MAPAMA, Ministerio de Agricultura y Pesca, Alimentación y Medio Ambiente. (2014). Guía de Gestión Integrada de Plagas: Olivar. 181 págs. Ministerio de Agricultura y Pesca, Alimentación y Medio Ambiente, Secretaría General Técnica, Centro de Publicaciones. Madrid.

[CR37] Nave, A., Gonçalves, F., Oliveira, I., Campos, M., & Torres, L. (2021). Does natural vegetation from olive groves benefit the olive moth, *Prays oleae*?. *Journal of Applied Entomology*, *145*(5), 406–416. 10.1111/jen.12859

[CR38] Ortega M, Pascual S (2014). Spatio-temporal analysis of the relationship between landscape structure and the olive fruit fly *Bactrocera oleae* (D iptera: T ephritidae). Agricultural and Forest Entomology.

[CR39] Ortega M, Pascual S, Rescia AJ (2016). Spatial structure of olive groves and scrublands affects *Bactrocera oleae* abundance: A multi-scale analysis. Basic and Applied Ecology.

[CR40] Ortega M, Sánchez-Ramos I, González-Núñez M, Pascual S (2018). Time course study of *Bactrocera oleae* (D iptera: T ephritidae) pupae predation in soil: The effect of landscape structure and soil condition. Agricultural and Forest Entomology.

[CR41] Ortega M, Moreno N, Fernández CE, Pascual S (2021). Olive landscape affects Bactrocera oleae abundance, movement and infestation. Agronomy.

[CR42] Paredes D, Cayuela L, Campos M (2013). Synergistic effects of ground cover and adjacent vegetation on natural enemies of olive insect pests. Agriculture, Ecosystems & Environment.

[CR43] Paredes D, Karp DS, Chaplin-Kramer R, Benítez E, Campos M (2019). Natural habitat increases natural pest control in olive groves: Economic implications. Journal of Pest Science.

[CR44] Pérez-Álvarez R, Nault BA, Poveda K (2019). Effectiveness of augmentative biological control depends on landscape context. Scientific Reports.

[CR45] Perović DJ, Gurr GM, Raman A, Nicol HI (2010). Effect of landscape composition and arrangement on biological control agents in a simplified agricultural system: A cost–distance approach. Biological Control.

[CR46] Plata Á, Moreno-Chocano J, Manjón-Cabezas J, Campos M, Paredes D (2019). Influencia de los hábitats naturales adyacentes en la plaga del olivo *Prays oleae*. Ecosistemas: Revista Cietifica y Tecnica De Ecologia y Medio Ambiente.

[CR47] Potts SG, Biesmeijer JC, Kremen C, Neumann P, Schweiger O, Kunin WE (2010). Global pollinator declines: Trends, impacts and drivers. Trends in Ecology & Evolution.

[CR48] Prasifka JR, Heinz KM, Minzenmayer RR (2004). Relationships of landscape, prey and agronomic variables to the abundance of generalist predators in cotton (Gossypium hirsutum) fields. Landscape Ecology.

[CR49] Rejili M, Fernandes T, Dinis AM, Pereira JA, Baptista P, Santos SA, Lino-Neto T (2016). A PCR-based diagnostic assay for detecting DNA of the olive fruit fly, *Bactrocera oleae*, in the gut of soil-living arthropods. Bulletin of Entomological Research.

[CR50] Rempoulakis1, P. & Nestel, D. (2011). Dispersal ability of marked, irradiated olive fruit flies [Bactrocera oleae (Rossi) (Diptera: Tephritidae)] in arid regions. *Journal of Applied Entomology 136*, 171–180. 10.1111/j.1439-0418.2011.01623.x

[CR51] Rescia AJ, Sanz-Cañada J, Bosque-González I (2017). A new mechanism based on landscape diversity for funding farmer subsidies. Agronomy for Sustainable Development.

[CR52] Rescia AJ, Ortega M (2018). Quantitative evaluation of the spatial resilience to the B. oleae pest in olive grove socio-ecological landscapes at different scales. Ecological Indicators.

[CR53] Reynolds C, Fletcher RJ, Carneiro CM, Jennings N, Ke A, LaScaleia MC, Lukhele MB, Mamba ML, Sibiya MD, Austin JD, Magagula CN, Mahlaba T, Monadjem A, Wisely SM, McCleery RA (2018). Inconsistent effects of landscape heterogeneity and land-use on animal diversity in an agricultural mosaic: A multi-scale and multi-taxon investigation. Landscape Ecology.

[CR54] Richard Y, Armstrong DP (2010). Cost distance modelling of landscape connectivity and gap-crossing ability using radio-tracking data. Journal of Applied Ecology.

[CR55] Rusch A, Chaplin-Kramer R, Gardiner MM, Hawro V, Holland J, Landis D, Thies C, Tscharntke T, Weisser WW, Winqvist C, Woltz M, Bommarco R (2016). Agricultural landscape simplification reduces natural pest control: A quantitative synthesis. Agriculture, Ecosystems & Environment.

[CR56] Schmidt MH, Thies C, Nentwig W, Tscharntke T (2008). Contrasting responses of arable spiders to the landscape matrix at different spatial scales. Journal of Biogeography.

[CR57] Singleton, P. H., Gaines, W. L., & Lehmkuhl, J. F. (2002). Landscape permeability for large carnivores in Washington: A geographic information system weighted-distance and least-cost corridor assessment. Res. Pap. PNW-RP-549. Portland, OR: US Department of Agriculture, Forest Service, Pacific Northwest Research Station. 89 p. 10.2737/PNW-RP-549

[CR58] Sivakoff FS, Rosenheim JA, Hagler JR (2012). Relative dispersal ability of a key agricultural pest and its predators in an annual agroecosystem. Biological Control.

[CR59] Thorbek P, Bilde T (2004). Reduced numbers of generalist arthropod predators after crop management. Journal of Applied Ecology.

[CR60] Tscharntke T, Klein AM, Kruess A, Steffan-Dewenter I, Thies C (2005). Landscape perspectives on agricultural intensification and biodiversity–ecosystem service management. Ecology Letters.

[CR61] Tscharntke T, Tylianakis JM, Rand TA, Didham RK, Fahrig L, Batáry P, Bengtsson J, Clough Y, Crist TO, Dormann CF, Ewers RM, Frund J, Holt RD, Holzschuh A, Klein AM, Kleijn D, Kremen C, Landis DA, Laurance W, Westphal C (2012). Landscape moderation of biodiversity patterns and processes-eight hypotheses. Biological Reviews.

[CR62] Tscharntke T, Karp DS, Chaplin-Kramer R, Batáry P, DeClerck F, Gratton C, Hunt L, Ives A, Johsson M, Larsen A, Martin EA, Martínez-Salinas A, Meehan TD, O´Rourke, M., Poveda, K., Rosenheim, J. A., Rusch, A., Schellhorn, N., Wanger, T. C., Wratten, S., & Zhang, W.  (2016). When natural habitat fails to enhance biological pest control–Five hypotheses. Biological Conservation.

[CR63] Van Leusen, P. M. (2002). Pattern to process: Methodological investigations into the formation and interpretation of spatial patterns in archaeological landscapes (Doctoral dissertation, University of Groningen).

[CR64] Vanbergen AJ, Initiative TIP (2013). Threats to an ecosystem service: Pressures on pollinators. Frontiers in Ecology and the Environment.

[CR65] Vermeulen SJ, Campbell BM, Ingram JS (2012). Climate change and food systems. Annual Review of Environment and Resources.

[CR66] Villa, M., Santos, S. A., Sousa, J. P., Ferreira, A., da Silva, P. M., Patanita, I., Patania, I., Ortega, M., Pascual, S., & Pereira, J. A. (2020). Landscape composition and configuration affect the abundance of the olive moth (*Prays oleae*, Bernard) in olive groves. *Agriculture, Ecosystems & Environment*, 294, 106854. 10.3390/insects12010046

[CR67] Wang, X. G., Johnson, M. W., Daane, K. M., Nadel, H. (2009). High summer temperatures affect the survival and reproduction of olive fruit fly (Diptera: Tephritidae). *Environmental Entomology, 38*, 1496–1504. 10.1603/022.038.0518 10.1603/022.038.0518 19825305

[CR68] Wang LA, Goonewardene Z (2004). The use of MIXED models in the analysis of animal experiments with repeated measures data. Canadian Journal of Animal Science.

[CR69] Wu P, Axmacher JC, Li X, Song X, Yu Z, Xu H, Tscharntke T, Westphal C, Liu Y (2019). Contrasting effects of natural scrubland and plantation forests on bee assemblages at neighboring apple orchards in Beijing, China. Biological Conservation.

[CR70] Zhang W, Ricketts TH, Kremen C, Carney K, Swinton SM (2007). Ecosystem services and dis-services to agriculture. Ecological Economics.

